# Oxylipins as Potential Regulators of Inflammatory Conditions of Human Lactation

**DOI:** 10.3390/metabo12100994

**Published:** 2022-10-20

**Authors:** Rachel E. Walker

**Affiliations:** Department of Nutritional Sciences, The Pennsylvania State University, University Park, PA 16802, USA; rew5009@psu.edu; Tel.: +1-(814)-863-0806

**Keywords:** lactation, oxylipins, inflammation, milk fat

## Abstract

Chronic low-grade inflammation can be associated with obesity or subclinical mastitis (SCM), which is associated with poor infant growth in low- to middle-income country settings. It is unknown what physiological mechanisms are involved in low milk supply, but our research group has shown that mothers with low milk supply have higher inflammatory markers. Studies investigating oxylipin signaling have the potential to help explain mechanisms that mediate the impacts of inflammation on milk production. Animal studies have reported various elevated oxylipins during postpartum inflammation, mastitis, and mammary involution in ruminant models. Several investigations have quantified oxylipins in human milk, but very few studies have reported circulating oxylipin concentrations during lactation. In addition, there are technical considerations that must be addressed when reporting oxylipin concentrations in human milk. First, the majority of milk oxylipins are esterified in the triglyceride pool, which is not routinely measured. Second, total milk fat should be considered as a covariate when using milk oxylipins to predict outcomes. Finally, storage and handling conditions of milk samples must be carefully controlled to ensure accurate milk oxylipin quantitation, which may be affected by highly active lipases in human milk.

## 1. Introduction

The numerous benefits of breastfeeding for infant health have been well-documented and include decreased risk of necrotizing enterocolitis in preterm infants as well as decreased risk of otitis media, acute diarrheal disease, and asthma in all infants [1,2]. Growing evidence also indicates that breastfeeding results in maternal health benefits, including reduced risk of breast cancer, diabetes, and cardiovascular disease in later life [3,4,5,6,7]. In addition to the profound health benefits for mothers and infants, higher breastfeeding rates in the United States and globally would lower the negative economic impacts of high medical costs [8] and reduce the inherent food waste and energy usage associated with infant formula production and feeding [9,10].

Despite the known benefits of breastfeeding, many families struggle to meet their breastfeeding goals, and only 25% of American mothers meet the recommendations of exclusive breastfeeding for 6 months and continued breastfeeding through 1–2 years of age [11]. In the US national consumer opinion panel survey taken in 2008, insufficient milk production, slow infant growth, and delayed onset of milk production were listed among the top reasons that families stopped breastfeeding earlier than desired, all potential indicators of physiologically low milk production. In fact, only 40% of mothers in this survey felt that they were able to breastfeed as long as they desired [12]. In low- and middle-income countries, low milk supply can be devastating for infant health due to unreliable access to infant formula and clean water [13,14]. Given the importance of this topic, it is surprising that very little is known about the physiological factors impacting low milk production in humans [15], and even less is known about what interventions might be effective [16]. Ongoing work in our research group has shown that poor metabolic health, inflammation, and obesity are associated with low milk production, but mechanisms explaining these observations have yet to be fully elucidated [17,18]. There is a great opportunity and potential in the field of lipidomics to address this gap and improve our understanding of human lactation physiology. 

Oxylipins are a diverse class of enzymatically produced specialized signaling molecules derived from polyunsaturated fatty acids (PUFA). Both omega-3 and omega-6 oxylipins are involved in the initiation and resolution of inflammatory processes [19] and have been used as biomarkers of several disease states [20]. Both pharmaceutical and dietary interventions have been reported to alter circulating oxylipin profiles, indicating that oxylipins may be involved in mediating the beneficial effects of anti-inflammatory treatments [20]. Oxylipin content in human milk could be an important biomarker of maternal health and have profound impacts on health outcomes and inflammatory conditions in breastfed infants. In fact, there is a growing body of research investigating the oxylipin content of human milk, showing promise for future studies to improve our understanding of this complex biological system. In particular, there is interest in quantifying the concentrations of specialized pro-resolving lipid mediators (SPMs) in human milk because of their potential to reduce disease severity in infants [21,22,23]. SPMs include the fatty acid metabolite classes of resolvins, protectins, lipoxins, and maresins, all PUFA metabolites that participate in the resolution of the inflammatory response [24]. The purpose of this narrative review is to summarize the current knowledge of oxylipin signaling during lactation, with a focus on human research. This review will also highlight the important knowledge gaps and technical challenges unique to studying oxylipins in human milk.

## 2. Inflammatory Disorders of Lactation

Inflammatory breast conditions during lactation are common, especially in the first few weeks after birth, and can include milk stasis, mastitis, and breast abscess. Mastitis occurs in up to 33% of lactating women in some populations [25], and is characterized by inflammation of the breast when intraductal pressure causes damage to the alveolar tight junctions and encroachment of milk into connective tissue [26]. This is usually caused by ineffective milk removal during the first few weeks of lactation and is especially common in primiparous mothers. This inflammation is often, but not always, accompanied by infection, usually involving *Staphylococcus* bacterial species. One of the most profound outcomes of mastitis is a significant drop in milk production, leading many mothers to supplement with infant formula or stop breastfeeding earlier than intended [25].

Traditionally, mastitis is considered an acute condition to be treated by improved emptying of milk from the breast, rest, fluid intake, non-steroidal anti-inflammatory medications, and sometimes antibiotic treatment [26]. However, in several low- and middle-income country settings, a chronic form of breast inflammation has been identified, known as subclinical mastitis (SCM; Defined as Na:K ratio >0.6 or >1) [27,28,29]. This chronic inflammation may be a result of insufficient inflammatory resolution following infection or other diseases. Although SCM does not result in clinical symptoms, this low level of inflammation in the breast loosens the tight junctions, resulting in a higher Na:K ratio in milk as well as elevated inflammatory cytokine concentrations [27,30,31]. The outcomes of subclinical breast inflammation are subtle, but studies have found reductions in infant growth [29,32,33,34] and a higher risk of human immunodeficiency virus transmission in breastmilk [28]. Although rigorous measures of milk production volume are rare, reduced infant growth in exclusively breastfeeding infants may indicate that a mother is experiencing lower milk production or lower milk energy density. More work is needed to explain this link between subclinical breast inflammation and poorer infant outcomes. 

In high-resource settings, repeated infection resulting in chronic inflammation is rare, but another source of low-grade chronic inflammation, obesity, is common. Mothers with obesity are less likely to meet breastfeeding recommendations and achieve their breastfeeding goals [35,36]. Obesity is also associated with delayed onset of milk production, a sign of physiological dysfunction in the mammary gland [37]. We have found that poor metabolic health, obesity, and inflammatory markers are all associated with low milk production despite intentions to exclusively breastfeed and frequent breast emptying [17,18]. In the case of low-grade inflammation from both obesity and SCM, poor milk production may result. However, targeted therapies and lifestyle interventions to improve milk volume are currently lacking [38,39]. For most mothers, the best option to build milk supply is frequent breast emptying by pumping, but this strategy is insufficient for a segment of mothers [17,40].

**Figure 1:**
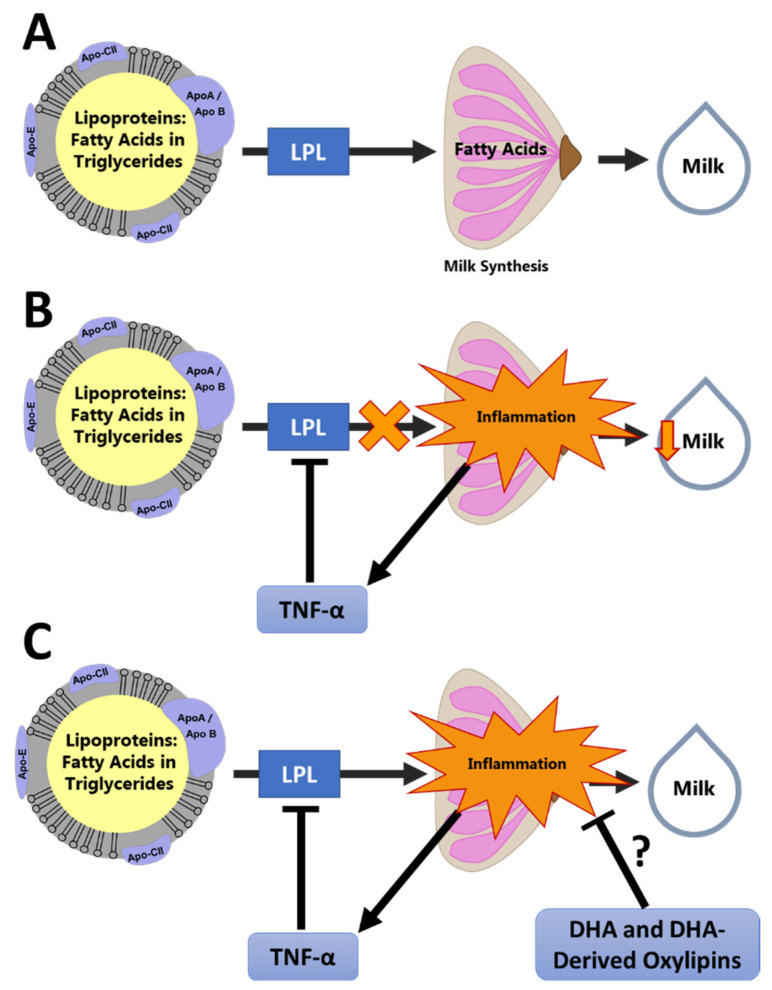
Conceptual Model of the Effect of Inflammation on Milk Production. This conceptual framework illustrates how oxylipins may be involved in resolution of mammary inflammation, leading to improved milk synthesis: (**A**) Fatty acids are oxidized by the mammary gland for energy and used as substrate for synthesis of the milk fat globule. This process requires uptake of fatty acids from the circulating lipoproteins by LPL. (**B**) Inflammation causes suppression of LPL by TNF-α, reducing fatty acid availability in the mammary gland and reducing milk synthesis. (**C**) We hypothesize that pro-resolving oxylipins, such as those derived from docosahexaenoic acid (DHA), reduce mammary inflammation and promote normal fatty acid uptake and milk synthesis.

Our group has developed a conceptual model linking inflammation to low milk supply (Figure 1). In this conceptual model, unresolved chronic inflammation leads to elevated inflammatory cytokines, such as tumor necrosis factor α (TNF-α). TNF-α is a potent inhibitor of gene expression for lipoprotein lipase (LPL) [41,42], which is critical for the uptake of fatty acids by the mammary gland [43]. Therefore, inflammation may result in a reduced availability of fatty acids to the mammary gland to be used for energy and as a substrate for milk fat globule synthesis. This is supported by our observation that mothers with low milk production and inflammation also have no association between blood and milk fatty acid profiles, indicating poor mammary fatty acid uptake [18]. Additionally, during inflammation, glucose uptake is markedly increased in the immune cells, redirecting energy availability away from the mammary gland to be used by the immune system [44].

Given this conceptual model, proper resolution of the inflammatory response following injury or infection is vital to ensure energy and substrate availability for milk synthesis. Oxylipin products of the cyclooxygenase (COX), lipoxygenase (LOX), and cytochrome P 450 epoxygenase (CYP 450_Ep_) pathways are involved in the inflammatory response in complex and diverse ways [19,20], acting as both initiators [45,46] and resolvers [47] of the immune response. Epoxide products of the CYP 450_Ep_ pathway have multiple beneficial effects including anti-inflammatory, anti-hypertensive, and analgesic effects [48], while the LOX products of omega-3 fatty acids are precursors to the SPM classes [47]. With their role in regulating inflammation, there is great promise in using oxylipins to investigate the underlying mechanisms linking low milk production with inflammatory conditions of lactation. However, the literature on oxylipins during lactation is limited, especially in humans, and there are several important technical challenges that must be considered when measuring oxylipins in human milk. There is a sizable body of evidence documenting the presence of prostaglandins from the COX pathway in human milk using immunoassays [49,50,51], and linking these with cytokine concentrations [49] and preterm delivery [51], among other outcomes. This narrative review will focus primarily on evidence from more recent lipidomic investigations that include the oxylipin products of the LOX and CYP 450_Ep_ pathways, since these pathways represent the primary knowledge gaps in the literature and produce the precursors of SPMs. Oxylipins referred to in this narrative review are listed by parent fatty acid in Table 1.

These oxylipins have been routinely measured in circulation, but many can also be measured in human milk. Linoleic acid oxylipin products are particularly abundant in milk [52,53], and the highly bioactive SPM classes and their HDoHE, HEPE, and HETE precursors have also been characterized [21] as well as several other LOX and CYP 450_Ep_ pathway products of ALA, ArA, EPA, and DHA [52].

## 3. Oxylipins during Lactation

### 3.1. Milk Oxylipins

There is considerable and growing interest in investigating the profile of bioactive components in milk, including oxylipins and other bioactive lipids. The National Institutes of Health have recently recognized the need for research treating mother, infant, and milk as a complex biological system with all three members of the triad affecting outcomes in the others [54]. With this increased interest, studies measuring and reporting multiple oxylipin concentrations in human milk are beginning to appear in the literature (Table 2). Reflecting the early stage of this field of research, several studies reporting oxylipin content in both human and bovine milk have focused on methodological considerations [52,53,55,56,57,58,59].

Some of the earliest milk oxylipin studies reported prostaglandins and leukotrienes in human milk at high concentrations [49,50,51]. These early studies utilized immunoassays and were limited by antibody availability at the time. More recent studies can take advantage of lipidomic platforms to measure a large number of oxylipin species in a single study. Weiss and colleagues measured detectable levels of non-esterified anti-inflammatory and pro-resolving lipid mediators, including leukotriene B, lipoxins, and resolvins, in human milk during early lactation. In addition, these researchers demonstrated that DHA and its non-esterified hydroxy product, 17-HDoHE, decrease with time over the course of lactation [21]. Robinson et al. similarly found a reduction across lactation in DHA and ArA in an analysis of preterm milk over the first four weeks of lactation. However, they found that non-esterified oxylipin products of DHA, such as 17-HDoHE, did not change over the same time course. In this study, concentrations for several pro-resolving lipid mediator products of DHA were imputed due to levels below the limit of quantitation [23]. 

Arnardottir and colleagues reported the profile of pro-resolving mediators measured in commercially available human donor milk and showed that this profile differed between donor milk from healthy donors and milk donated from mastitis-affected mammary glands. In addition, they showed treatment with human milk lipid mediator isolates reduced inflammatory resolution time in induced murine peritonitis and improved human macrophage function [22]. From this experiment, the potential for benefits to infant health from human milk pro-resolving oxylipins is clear and highly exciting. However, the evidence for human health outcomes related to oxylipins is quite limited. One study reported lipidomic differences in human milk from mother–infant dyads with slow growth trajectories compared with fast growth trajectories. Significant differences were observed in some prostaglandin, leukotriene, and thromboxane concentrations [60]. 

Oxylipin concentrations have been reported in milk from several animal models as well. In lactating dairy cows, most milk oxylipins steadily increase in concentration from early to late lactation [63]. Bovine mastitis is associated with changing linoleic acid oxylipin profiles in both mammary tissue and milk [62]. Mavangira and colleagues observed increased LOX pathway HODEs and HETEs, as well as CYP 450_Ep_ pathway EpOMEs, DiHETrEs, and DiHETEs in milk from cows with coliform mastitis compared with healthy controls. This study also found that 9-HETE, which is not produced enzymatically and is a marker of lipid autoxidation, was elevated during mastitis [61]. Oxylipins in general, and 13-HODE in particular, increase in milk during late bovine lactation, potentially signaling the initiation of mammary involution and apoptosis of the mammary epithelial cells [63]. Taken together, this indicates that elevated oxylipin concentrations in bovine milk may be biomarkers of mastitis-related inflammation or of the onset of mammary apoptosis related to involution. 

In contrast to the bovine studies, higher oxylipin levels in swine milk were associated with beneficial piglet outcomes in one study. Llaurado-Calero and colleagues found that fish oil was associated with increased EPA and DHA content in colostrum and milk as well as improved piglet survival and growth. Fish oil feeding increased concentrations of most oxylipins, even those derived from 18 carbon and omega-6 PUFA, but not surprisingly, the largest differences were measured in the hydroxy and di-hydroxy products of DHA and EPA [64]. Differences in outcomes between bovine and swine experiments suggest that the ‘positive’ or ‘negative’ effects of oxylipins in the mammary gland may depend on species and environmental context.

### 3.2. Circulating Oxylipins

Milk oxylipins can provide a window into inflammatory and pro-resolving signaling in the mammary gland, but systemic inflammation and metabolism may also be impacting milk synthesis. Therefore, systemic oxylipin profiles measured in the circulation may also be important in understanding the impact of inflammatory processes on human lactation. However, only a single report has been found reporting circulating oxylipin profiles in lactating humans [65] (Table 3), and that study reported vitamin A status, not lactation or breastfeeding measures, as outcomes. A few studies have reported circulating oxylipins during pregnancy in humans [66,67,68,69,70], showing potential associations with infant growth [70] and pre-term birth [67,69], but have not investigated breastfeeding outcomes. More work is needed to characterize the systemic oxylipin profile in human circulation during lactation.

The production animal literature is a deeper knowledge source for circulating oxylipins during lactation, although data are limited even in animal models. Some studies have characterized changes in circulating oxylipins throughout stages of reproduction. Kuhn and colleagues found that plasma 20-HETE was elevated in the early post-partum, then decreased, while 9(10)-DiHOME showed the opposite pattern and 13-oxo-ODE decreased steadily across lactation in dairy cows [63]. In contrast, plasma oxylipin profiles generally remain unchanged across a single bovine diestrus phase in lactating dairy cows [73]. 

The linoleic acid product of the 15-LOX activity, 13-HpODE is elevated in mastitis and can stimulate apoptosis in bovine mammary epithelial cells, while 13-HODE and 13-oxo-ODE have a neutral or possibly anti-inflammatory effect [62,74]. Putman et al. similarly found significant increases in many circulating COX, LOX, and CYP 450_Ep_ pathway oxylipins associated with involution during the dry-off period in dairy cows [71]. Mavangira and colleagues showed that circulating oxylipin profiles were altered in dairy cows with mastitis, but these changes were generally less pronounced than changes in milk oxylipin concentrations [61]. Postpartum ewes treated with the non-steroidal anti-inflammatory medication meloxicam had reduced inflammation in the early post-partum period, but changes in the plasma oxylipin profile were modest. There was a significant reduction in the CYP 450_Ep_ pathway oxylipin, 9(10)-DiHOME [72]. Taken together, these animal studies generally replicate the findings from animal studies of milk oxylipins in which oxylipins appeared to be biomarkers of mammary inflammation or involution. However, the translation of lactation physiology from ruminant data to human applications is problematic for several reasons. Ruminant lipid metabolism is greatly impacted by the microbial populations in the rumen that alter many ingested PUFA before they are absorbed into the bloodstream [75]. In addition, temporal changes across lactation in fatty acid composition are much different in humans compared with bovine milk. For example, milk omega-3 fatty acid content generally decreases over time throughout a human lactation [21,23], but increases throughout the course of a bovine lactation [63].

## 4. Oxylipins and Chronic Low-Grade Inflammation during Lactation

Both obesity and SCM are examples of commonly occurring conditions associated with chronic low-grade inflammation. Currently, there is a lack of evidence for the expected role of circulating and mammary oxylipin signaling in mediating the effects of low-grade inflammation on lactation. However, at least one study has reported oxylipin profile differences with obesity during pregnancy [69], and milk fat composition is known to be altered with obesity. Borkowski and colleagues investigated the association between circulating non-esterified oxylipins and pre-term birth stratified by obesity status. They found that higher concentrations of prenatal circulating lipoxygenase and autoxidation products of ArA, EPA, and DHA were associated with pre-term birth in mothers with obesity but not in mothers with normal weight [69]. This suggests that lipid-mediated inflammatory signaling may be associated with pre-term birth in mothers with obesity, but no inflammatory markers were reported.

Total milk fat content does not appear to be altered with SCM [76], but several studies have reported differences in total milk fat content associated with obesity and BMI. A positive association between maternal BMI and milk fat content has been reported in cohorts in Poland [77], the United States [78,79], Guatemala [80], and Canada [81]. Milk fatty acid profile also changes with obesity. Specifically, milk from mothers with obesity and/or overweight has been reported to have higher saturated fatty acids [81,82], lower monounsaturated fatty acids [81,82], lower omega-3 PUFA [81,83], lower omega-6 PUFA [81], and higher omega-6:omega-3 ratio [83] compared with milk from mothers who are lean. With these differences in milk fatty acid content, especially differences in PUFA profile, it would be expected that there are also differences in milk oxylipin concentrations. Milk oxylipin profiles will undoubtedly have biological implications for infant health, growth, and development, and could also provide an important window into the inflammatory signaling environment of the mammary gland.

## 5. Gaps in Knowledge

The current evidence suggests that oxylipins have a strong potential to be potent mediators of inflammatory processes in the human mammary gland during lactation. However, several important large gaps remain in the current literature. As outlined before, there is a lack of studies that report circulating maternal oxylipins during lactation, which could elucidate the role of systemic inflammatory signals in mammary gland inflammation and milk synthesis. In addition, there is a lack of mechanistic studies linking chronic low-grade inflammation to milk synthesis. For example, although LPL is highly expressed in the mammary gland [43], data are missing in the literature about how mammary LPL expression is regulated during inflammation. Because there is a gap in the understanding of the physiological mechanisms of low milk production, there is also a dire lack of effective strategies to treat low milk production in humans. A better understanding of oxylipin biology in the mammary gland and systemic oxylipins during lactation may lead to the identification of therapeutic targets.

Another gap in the current literature available on milk oxylipins is the lack of data in the esterified pool. Milk contains abundant esterified fatty acids and oxylipins in the milk fat globule triglycerides [52,53]. However, most studies examining human milk oxylipin profiles have exclusively measured the non-esterified pool [21,22,55,57,59,60]. Although non-esterified oxylipins are the primary molecules available for bioactive functions, those esterified in triglycerides and phospholipids can be made available upon exposure to lipase activities. Esterified oxylipins in triglycerides would be made readily available for absorption within the infant’s gut through the combined action of LPL and bile salt-stimulated lipase in human milk [84] as well as the infant’s own intestinal lipases. Therefore, esterified oxylipins are an important potential pool of bioactive oxylipins for the infant and should not be ignored. Future studies should consider measuring total and esterified oxylipins in addition to the non-esterified pool to fully represent the potential bioactive signals present in milk. This can easily be done by including a hydrolysis step prior to solid phase extraction when preparing samples for oxylipin analysis [52,53].

## 6. Technical Challenges in Measuring Milk Oxylipins

There are several important technical factors to consider when measuring oxylipins in milk, including:

1. The total milk fat concentration;
2. Potential matrix effects of milk affecting the extraction efficiency;
3. High lipase activity in human milk.

**Figure 2:**
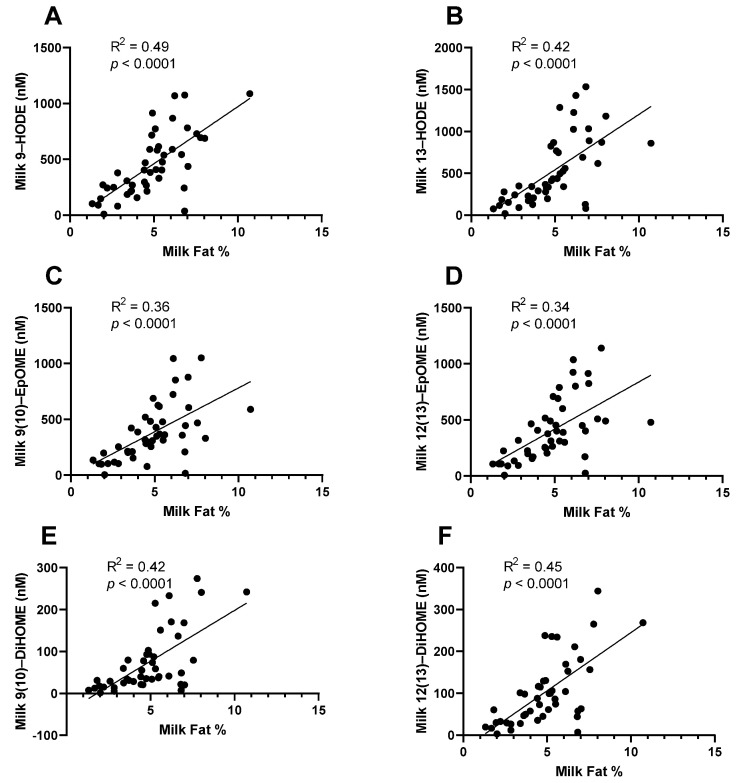
Total (esterified + non-esterified) oxylipins measured in whole milk are strongly correlated with total milk fat. Samples were collected as part of a case–control study of low milk supply. Most oxylipins, including the linoleic acid-derived oxylipins, (**A**) 9-HODE, (**B**) 13-HODE, (**C**) 9(10)-EpOME, (**D**) 12(13)-EpOME, (**E**) 9(10)-DiHOME, and (**F**) 12(13)-DiHOME, were strongly correlated with the total milk fat measured in the sample (*n* = 46).

When reporting milk fatty acid profile, data are usually presented as the weight% of total fatty acids. This is partly due to the fact that absolute concentration is mostly determined by the total milk fat content of milk, which is highly variable [85]. It is likely that the same is true of milk oxylipins, with the absolute concentration being strongly dependent on the total milk fat content. In our research group, we found that the concentration of total oxylipins (esterified + non-esterified) measured in whole milk is strongly correlated with milk fat% (previously unpublished data, Figure 2). Therefore, it is likely that differences observed between groups are more strongly explained by differences in milk fat content than by differences in oxylipin signaling pathways within the mammary gland. It is unknown how non-esterified oxylipins may be related to total milk fat%, but this should be investigated. Investigators should consider how total milk fat may be affecting their results. For example, infant growth is impacted by milk fat content in exclusively breastfeeding infants, since milk fat is the primary determinant of variation in the energy density of milk. Investigators reporting infant growth outcomes related to milk oxylipins should investigate if those outcomes could be primarily explained by total milk fat, or if the milk oxylipins are acting independently, and total milk fat should be considered as a covariate in statistical models.

A second technical concern is low extraction efficiency, as observed by Gouveia-Figueira and colleagues. In their report, they found that using protocols optimized for detecting oxylipins in plasma resulted in significantly lower extraction efficiencies in human milk compared with other types of biological samples [59]. Some potential explanations for this could be because of the very high lipid content in milk or the higher pH of the milk matrix. Therefore, groups extracting and quantitating milk oxylipins should report extraction efficiency and consider methods to optimize their recovery, such as utilizing double or triple extractions or using buffers to optimize pH for extraction of the acidic non-esterified oxylipins.

At least two studies have reported that sampling and processing conditions (storage time, storage temperature, and pasteurization) of human milk change oxylipin concentrations profoundly [55,57], even though fatty acid concentrations stay relatively stable [56]. Of specific concern, Wu and colleagues found significant increases in oxylipin concentrations when samples were stored for 3 months, even at −80 °C [57]. However, evidence for proper storage and handling of human milk for oxylipin analysis is extremely limited [56].

**Figure 3:**
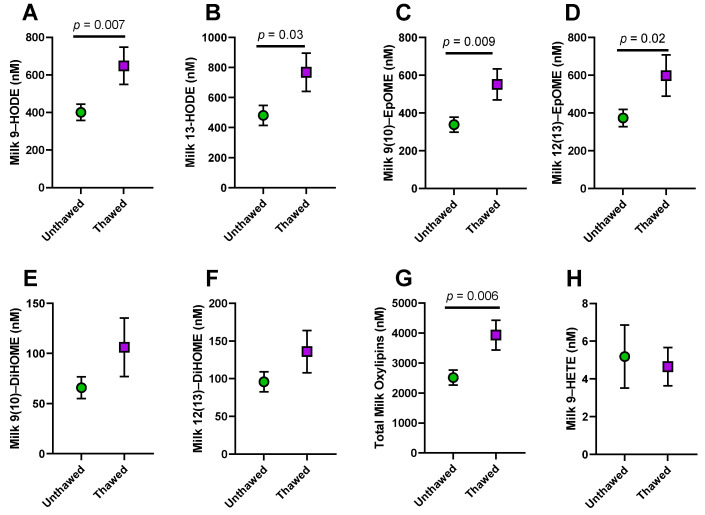
Differences in milk total oxylipins between unthawed samples and samples with previous freeze–thaw cycles. Samples were collected as part of a case–control study of low milk supply. In this study, unthawed samples were not available for all subjects, so we tested for differences with previous freeze–thaw cycles. Total oxylipin (esterified + non-esterified) concentrations were higher with previous thawing for (**A**) 9-hydroxy octadecadienoic acid (9-HODE), (**B**) 13-HODE, (**C**) 9(10)-epoxy octadecamonoenoic acid (9(10)-EpOME), (**D**) 12(13)-EpOME, (**E**) 9(10)-dihydroxy octadecamonoenoic acid (9(10)-DiHOME), (**F**) 12(13)-DiHOME, and (**G**) Total Oxylipins, although the difference was not statistically significant for DiHOMEs. In contrast, (**H**) 9-hydroxy eicosatetraenoic acid (9-HETE), a marker of autoxidation, was not different from previous thawing. Unthawed, *n* = 36; Previously thawed, *n* = 10. Differences assessed by *t*-test. Data graphed as mean ± SEM.

Our research group had access to milk samples from a case–control study [17] of low milk supply which had been stored for 4–7 years at −80 °C. Some of these samples had not been previously thawed, while others had been thawed multiple times. In order to determine which samples should be considered for oxylipin analysis, we analyzed total oxylipin (esterified + non-esterified) concentrations for differences between the unthawed samples and those that had been previously thawed and found that previously thawed samples had significantly higher concentrations of many oxylipins (Previously unpublished data; Figure 3A–G).

It is possible that increased oxylipin concentration with thawing is due to autoxidation of the milk PUFA. We examined if there was a difference between the number of freeze–thaw cycles and 9-HETE concentrations, a marker of autoxidation. In our data, there was no difference in 9-HETE concentrations by freeze–thaw cycles, suggesting that autoxidation is not the most likely explanation (Figure 3H). An alternative theory is that thawing activates LPL found abundantly in human milk as well as the lipolytic activity of the bacteria found in the milk microbiome [57]. LPL releases PUFA from the abundant triglycerides in the milk fat globule, providing a substrate for the enzymatic production of oxylipins. 

There is evidence for the theory that elevated oxylipins could be the result of lipolysis during storage and processing. Oxylipins are produced when COX, LOX, and CYP450_Ep_ enzymes incorporate oxygen atoms into non-esterified PUFA. Therefore, lipases increase the potential substrate for these oxylipin-producing enzymes to act upon. Human milk is very high in lipase enzymes, particularly LPL, leading to elevated non-esterified fatty acid content with prolonged storage [84,86,87]. This is particularly true when milk is stored unfrozen, but some lipase activity has been observed even with prolonged frozen storage at temperatures of −20 °C or higher [84]. Lipolysis is not generally considered a major factor in measuring fatty acid profile or even total triglyceride content, because the triglyceride content in the milk fat globule is extremely high. However, because of the low normal concentrations of oxylipins in human milk, even small increases in the substrate for the COX, LOX, or CYP450_Ep_ enzyme pathways would have profound impacts on the concentrations of these molecules.

Pasteurization also alters oxylipin concentrations, with different patterns when using different pasteurization techniques [55]. Increases in oxylipin concentrations during extended storage and processing could partially explain the detection of high concentrations of pro-resolving lipid mediators in some studies using donor milk [22] or milk stored for a significant time at −20 °C [21], while other studies were unable to detect them reliably [23]. Milk collection, storage, and handling procedures are critical in order to understand the biological concentrations of oxylipins produced by the mammary gland. At a minimum, all studies should report their sample handling and storage procedures, and studies intending to analyze milk oxylipins should be designed to minimize sample storage times, especially at temperatures higher than −70 °C.

## 7. Conclusions

There is strong potential to use oxylipins during lactation, both in milk and circulation, to identify mechanisms linking inflammation to lactation and breastfeeding outcomes. Specifically, mechanistic studies are needed to clarify the physiological link between obesity and inflammation with low milk supply. Oxylipins play important roles in initiating and resolving inflammation, and milk oxylipin concentrations may represent biomarkers of the inflammatory state of the mammary gland. DHA- and EPA-derived oxylipins may have particular potential in mediating the inflammatory resolution needed to return low-grade inflammation back to baseline. However, studies reporting oxylipin concentrations in milk must consider several technical complexities in their study design and interpretation. First, the oxylipin compartment measured should be considered. Although the non-esterified oxylipin pool is considered the most bioactive, the majority of oxylipins in milk are contained in the esterified pool as part of the milk fat globule triglycerides and phospholipids. Therefore, the esterified pool should be considered, especially when using oxylipins to predict infant outcomes, since esterified oxylipins will be made available to the infant during digestion and absorption of the milk fat. Second, milk oxylipins are strongly correlated with total milk fat. Therefore, when using milk oxylipins to predict and study specific outcomes, researchers must consider if the outcomes could be explained by total milk fat concentration and consider using milk fat content as a covariate in statistical models. Third, studies have shown that concentrations of milk oxylipins increase significantly with extended storage, especially when unfrozen. Preliminary evidence from our research group also shows that samples with multiple freeze–thaw cycles have significantly higher oxylipin concentrations compared with unthawed samples. Therefore, sample handling is an important variable that must be controlled in study designs using milk oxylipins. Future carefully designed studies are needed in this important topic area and have strong potential to improve outcomes for lactating mothers and their infants.

## Figures and Tables

**Table 1:** Cyclooxygenase (COX), lipoxygenase (LOX), and cytochrome P450 epoxygenase (CYP 450_Ep_) pathway oxylipins by parent fatty acid as discussed in this review.

Parent Fatty Acid	Enzymatic Pathway	Oxylipin Class (Abbreviation)
Linoleic Acid	LOX	Hydroxy octadecadienoic acids (HODEs)
	LOX	Hydroperoxy octadecadienoic acids (HpODEs)
	LOX	Oxo octadecadienoic acid (oxo-ODEs)
	LOX	Trihydroxy octadecamonoenoic acids (TriHOMEs)
	CYP 450_Ep_	Epoxy octadecamonoenoic acids (EpOMEs)
	CYP 450_Ep_	Dihydroxy octadecamonoenoic acids (DiHOMEs)
Alpha-linolenic Acid (ALA)	LOX	Hydroxy octadecatrienoic acids (HOTrEs)
	CYP 450_Ep_	Epoxy octadecadienoic acids (EpODEs)
	CYP 450_Ep_	Dihydroxy octadecadienoic acids (DiHODEs)
Arachidonic Acid (ArA)	COX	2-series prostaglandins (PG_2_s)
	COX	Keto prostaglandins (keto-PGs)
	COX	2-series thromboxanes (TX_2_s)
	LOX	Hydroxy eicosatetraenoic acids (HETEs)
	LOX	Oxo eicosatetraenoic acids (oxo-ETEs)
	LOX	4-series leukotrienes (LT_4_s)
	CYP 450_Ep_	Epoxy eicosatrienoic acids (EpETrEs)
	CYP 450_Ep_	Dihydroxy eicosatrienoic acids (DiHETrEs)
Eicosapentaenoic Acid (EPA)	COX	3-series prostaglandins (PG_3_s)
	COX	3-series thromboxanes (TX_3_s)
	LOX	Hydroxy eicosapentaenoic acids (HEPEs)
	LOX	3- and 5-series leukotrienes (LT_3,5_s)
	CYP 450_Ep_	Epoxy eicosatetraenoic acids (EpETEs)
	CYP 450_Ep_	Dihydroxy eicosatetraenoic acids (DiHETEs)
Docosahexaenoic Acid (DHA)	LOX	Hydroxy docosahexaenoic acids (HDoHEs)
	CYP 450_Ep_	Epoxy docosapentaenoic acids (EpDPEs)
	CYP 450_Ep_	Dihydroxy docosapentaenoic acids (DiHDPEs)
ArA, EPA, and DHA	LOX	Specialized Pro-Resolving Lipid Mediators (SPMs)

**Table 2:** Studies reporting lipidomic LOX and CYP450_Ep_ pathway oxylipin measurements in milk during lactation.

Study (First Author, Year)	Species	Sample Size	Oxylipin Pool	Oxylipin Classes Quantified	Sample Type	Study Oxylipin Outcomes
Weiss, 2013 [21]	Human	*n* = 30	Non-esterified	HETEs HEPEs HDoHEs LTs SPMs	Whole milk	17-HDoHE decreases across first 4 weeks of lactation in mothers of pre-term infants; Other pro-resolving mediator and precursor concentrations remain stable.
Gouveia-Figueira, 2015 [59]	Human	*n* = 3	Non-esterified	PGs HODEs EpOMEs DiHOMEs TXs HETEs oxo-ETEs EpETrEs DiHETrEs HEPEs	Whole milk	Many oxylipins were detectable in human milk, but extraction efficiency was lower in human milk than many other biological sample types.
Arnardottir, 2016 [22]	Human	*n* = 13	Non-esterified	PGs LTs TXs SPMs	Whole milk	Lipid mediator concentrations were recorded for donor milk from commercial supplier; Concentrations differed between donors with and without mastitis by principal components analysis.
Wu, 2016 [57]	Human	*n* = 1	Non-esterified	HODEs oxo-ODEs EpOMEs DiHOMEs TriHOMEs	Skim milk	Some oxylipin concentrations increase significantly with extended storage, especially when held for any period of time at -20oC or above.
Robinson, 2017 [23]	Human	*n* = 30	Non-esterified	PGs TXs LTs HETEs HEPEs HDoHEs SPMs	Whole milk	Oxylipin concentrations stayed stable across 4 weeks of lactation in mothers delivering full-term.
Alexandre-Gouabau, 2017 [60]	Human	*n* = 22	Non-esterified	PGs TXs LTs HETEs	Whole milk	10,11-dihydro-20-trihydroxy-LTB_4_ and PGs were associated with faster infant growth trajectories; 11-dihydro-2,3-dinor-TXB2 was associated with slower infant growth trajectories.
Pitino, 2019 [55]	Human	*n* = 17 (pooled)	Non-esterified	HODEs oxo-ODEs EpOMEs DiHOMEs HOTrEs PGs TXs HETEs oxo-ETEs EpETrEs DiHETrEs EpETEs DiHETEs EpDPEs	Whole milk	Oxylipin profiles were altered significantly with different pasteurization techniques.
Gan, 2020 [52]	Human	*n* = 5 (pooled)	Non-esterified Esterified neutral lipids Esterified phospholipids	HODEs oxo-ODEs EpOMEs DiHOMEs TriHOMEs HOTrEs HETEs oxo-ETEs EpETrEs DiHETrEs HEPEs EpETEs DiHETEs EpDPEs	Skim milk Cream layer Cell pellet	Non-esterified oxylipins were most abundant in skim milk, while esterified oxylipins were most abundant in cream layer and cell pellet; Over 90% of milk oxylipins were derived from linoleic acid.
Mavangira, 2015 [61]	Bovine	*n* = 24	Non-esterified	HODEs oxo-ODEs EpOMEs DiHOMEs PGs LTs HETEs EpETrEs DiHETrEs HDoHEs SPMs	Whole milk	Many oxylipin classes had increased concentrations in dairy cows with mastitis.
Ryman, 2015 [62]	Bovine	*n* = 8	Non-esterified	HODEs oxo-ODEs PGs TXs LTs HETEs oxo-ETEs SPMs	Whole milk	Milk from cows with S. uberis infection was higher in HODEs and 11-HETE.
Kuhn, 2017 [63]	Bovine	*n* = 36	Non-esterified	HODEs EpOMEs DiHOMEs HETEs DiHETEs EpDPEs SPMs	Whole milk	Most oxylipin concentrations increased with time across lactation.
Teixeira, 2021 [53]	Bovine	Pooled milk from storage tanks	Non-esterified Total	HODEs oxo-ODEs EpOMEs DiHOMEs HOTrEs HETEs EpETrEs DiHETrEs HEPEs EpETEs EpDPEs	Whole milk	Over 95% of oxylipins in milk were bound; Approximately 90% of milk oxylipins were linoleic acid-derived oxylipins; Lipid extraction followed by base hydrolysis in methanol was the best method for measuring total oxylipins.
Llaurado-Calero, 2021 [64]	Porcine	*n* = 36	Non-esterified	HODEs oxo-ODEs EpOMEs DiHOMEs TriHOMEs HOTrEs EpODEs DiHODEs PGs TXs HETEs EpETrEs DiHETrEs HEPEs DiHETEs HDoHEs DiHDPEs SPMs	Whole milk	Fish oil feeding improved piglet growth and survival; Fish oil feeding increased concentrations of most milk oxylipins.

DiHDPEs—Dihydroxy docosapentaenoic acids; DiHETEs—Dihydroxy eicosatetraenoic acids; DiHETrEs—Dihydroxy eicosatrienoic acids; DiHOMEs—Dihydroxy octadecamonoenoic acids; EpDPEs—Epoxy docosapentaenoic acids; EpETEs—Epoxy eicosatetraenoic acids; EpETrEs—Epoxy eicosatrienoic acids; EpOMEs—Epoxy octadecamonoenoic acids; HDoHEs—Hydroxy docosahexaenoic acids; HEPEs—Hydroxy eicosapentaenoic acids; HETEs—Hydroxy eicosatetraenoic acids; HODEs—Hydroxy octadecadienoic acids; HOTrEs—Hydroxy octadecatrienoic acids; LTs—Leukotrienes; oxo-ETEs—Oxo eicosatetraenoic acids; oxo-ODEs—Oxo octadecadienoic acid; PGs—Prostaglandins; SPMs—Specialized pro-resolving lipid mediators; TriHOMEs—Trihydroxy octadecamonoenoic acids; TXs—Thromboxanes.

**Table 3:** Studies reporting lipidomic LOX and CYP450_Ep_ pathway oxylipin measurements in circulation during lactation.

Study (First Author, Year)	Species	Sample Size	Oxylipin Pool	Oxylipin Classes Quantified	Sample Type	Study Oxylipin Outcomes
Johnson, 2022 [65]	Human	*n* = 10	Non-esterified	PGs LTs HETEs oxo-ETEs EpETrEs HEPEs DiHETEs SPMs	Plasma	Low plasma Vitamin A was associated with lower plasma concentrations of many oxylipins in lactating mothers.
Mavangira, 2015 [61]	Bovine	*n* = 24	Non-esterified	HODEs oxo-ODEs EpOMEs DiHOMEs PGs LTs HETEs EpETrEs DiHETrEs HDoHEs SPMs	Plasma	Multiple oxylipin classes had elevated concentrations in dairy cows with mastitis.
Kuhn, 2017 [63]	Bovine	*n* = 36	Non-esterified	HODEs EpOMEs DiHOMEs HETEs DiHETEs EpDPE SPMs	Plasma	20-HETE decreased and 9(10)-DiHOME increased from the periparturient to mid-lactation stage; 13-oxo-ODE decreased across the entire lactation.
Putman, 2019 [71]	Bovine	*n* = 10	Non-esterified	HODEs EpOMEs DiHOMEs HOTrEs PGs HETEs oxo-ETEs DiHETrEs DiHETEs HDoHEs EpDPEs DiHDPEs	Plasma	Many oxylipins spike in concentration around the time of dry-off and may be involved in the process of involution.
Olagaray, 2020 [72]	Ovine	*n* = 36	Non-esterified	HODEs oxo-ODEs EpOMEs DiHOMEs HOTrEs PGs TXs HETEs DiHETrEs HDoHEs EpDPEs DiHDPEs SPMs	Plasma	Treatment with meloxicam after birth reduced concentrations of some oxylipins, primarily 9(10)-DiHOME, in postpartum ewes.
King, 2021 [73]	Bovine	*n* = 30	Non-esterified	HODEs oxo-ODEs PGs LTs HETEs oxo-ETEs DiHETrEs HEPEs HDoHEs SPMs	Plasma Uterine flushing	Plasma oxylipins stay stable throughout early to late diestrous stage; Uterine oxylipins spike in late diestrous stage.

DiHDPEs—Dihydroxy docosapentaenoic acids; DiHETEs—Dihydroxy eicosatetraenoic acids; DiHETrEs—Dihydroxy eicosatrienoic acids; DiHOMEs—Dihydroxy octadecamonoenoic acids; EpDPEs—Epoxy docosapentaenoic acids; EpETrEs—Epoxy eicosatrienoic acids; EpOMEs—Epoxy octadecamonoenoic acids; HDoHEs—Hydroxy docosahexaenoic acids; HEPEs—Hydroxy eicosapentaenoic acids; HETEs—Hydroxy eicosatetraenoic acids; HODEs—Hydroxy octadecadienoic acids; HOTrEs—Hydroxy octadecatrienoic acids; LTs—Leukotrienes; oxo-ETEs—Oxo eicosatetraenoic acids; oxo-ODEs—Oxo octadecadienoic acid; PGs—Prostaglandins; SPMs—Specialized pro-resolving lipid mediators; TXs—Thromboxanes.

## Data Availability

The data presented in this study are openly available in FigShare at https://doi.org/10.6084/m9.figshare.21357363 (accessed on 8 September 2022).
